# Exchange rate instabilities during the Russia-Ukraine war: Evidence from V4 countries

**DOI:** 10.1016/j.heliyon.2024.e25476

**Published:** 2024-02-01

**Authors:** Florin Aliu, Jiří Kučera, Jakub Horák

**Affiliations:** School of Expertness and Valuation, Institute of Technology and Business, České Budějovice, Czech Republic

**Keywords:** Russia-Ukraine war, COVID-19 pandemics, V4 countries, Exchange rate parities, Gas

## Abstract

**Purpose:**

This study investigates the impact of the Russian Ruble on the Czech crown, Polish zloty, and Hungarian forint during the Russia-Ukraine war. The euro is used as a comparative base unit in the four exchange rate parities. The Euro was used since the Czech Republic, Hungary, Poland, and Russia maintain intensive economic relations with the Eurozone. At the same time, the Visegrad (V4) countries are geographically located in the European continent and are bordered by the Eurozone member states.

**Methods:**

The series stands in daily frequency and indicate the period from February 1, 2022, to February 1, 2023. To generate the results, the VAR impulse response function, variance decomposition, vector error correction model, and granger causality test were performed.

**Results:**

Even though Russia demanded that gas payments be made in Rubles, this fact did not affect the Czech crown, Polish zloty, and Hungarian forint. Due to the fact that gas payments for the V4 countries were agreed in Euros through German contractors. During this period, the strong influence of the Czech crown on the Polish zloty and the Hungarian forint is observed.

**Implications:**

From a policy perspective, the results provide indications for the national governments and regulatory bodies on the implications of the Russian ruble during this conflict. In short, our findings document that the instability of currency pairs is not only economic but also geopolitical. Energy dependence on autocratic states not only endangers national security but can set exchange rates in cardiac arrest. Moreover, the geographical proximity to the conflict zone tends to be decisive in the collapse of national currencies.

## 1 Introduction

The instabilities of the foreign exchange rate (FX) have permanently raised the interest of media, investors, and scholars. The exchange rate shocks and the factors driving them are at the center of academic debates and central banks' actions. The frequent spikes in the FX market generate insecurity in international trade and diminish portfolio performance [[1], [2], [3]]. The most traded currency pairs in FOREX are considered the American dollar (USD), the euro (EUR), the Canadian dollar (CAD), and the British pound (GBP). Due to the high trade volume that FOREX maintains, excesses on the system are adjusted quickly. International transactions are largely carried out in hard currencies such as the US Dollar (USD) and Euro (EUR) [4]. The economic and geopolitical weight that the US holds worldwide made the American dollar the most reliable currency. The outbreak of the COVID-19 pandemic placed global growth into negative territory [5] and disrupted currency exchanges. Due to supply chain difficulties, stringency measures, and monetary actions, currency pairs were profoundly affected [6]. Adding to this fact the restriction of the movement of people and goods caused central banks to intervene in their national currencies. To keep the sound financial system and avoid another meltdown, the central banks injected excessive liquidity. Until June 2020, the European Central Bank (ECB) added 1.3 trillion euros through quantitative easing policies [7]. The financial system was overloaded with excess liquidity and part of that money flowed into the equity market. Meanwhile, the Eurozone economic growth was contracting, while the equity markets were constantly on the rise [[8], [9], [10]]. The fragile economies with insufficient international reserves during this period experienced currency crashes. The work by Ref. [11] using dynamic VAR examined the long-term impact of COVID-19 on the Euro, Canadian Dollar, Japanese Yen, and Chinese Yuan. Their results indicate that 55 %–75 % of the movements are explained by their own lags. At the same time, exchange rates are prone to speculative elements and bubble formation. Enough evidence suggests that currency pairs perceived the shocks transmitted by COVID-19 and made them inefficient [12,13]. The estimated equilibrium of the FX pairs between the two currencies is subject to the supply and demand mechanism. Supply and demand are generally driven by international trade, capital movements, and speculative elements. In this article, we investigate the influence of the Russian Ruble in the three V4 currency pairs (Czech Republic, Poland, and Hungary) that are geographically close to the conflict zone. The Slovak Republic is not considered since it is a member of the Eurozone and has adopted euro since 2009. The results provide a modest contribution to the governments of these countries that were deeply dependent on Russian gas. Even though Russia demanded that these countries pay for the imported gas in rubles, this had no impact on their national currencies.

The Russian invasion of Ukraine on February 24, 2022, carried out devastating consequences for human life, civilian infrastructure, and worldwide inflation. Using the heteroscedasticity-based estimator, Tong [14] (2024) analyzed the impact of this war on 86 economies from January 2021 to November 2022. He showed that the implications appeared in the weakening of the pace of economic growth, accelerated inflation, and destabilized financial markets. As this article is being written, the armed conflict in Ukraine is intensifying and the number of refugees has exceeded seven million [15]. The outbreak of the COVID-19 pandemic and the subsequent Russian invasion of Ukraine created an unprecedented panic for the investors. This caused the central banks to become active in keeping inflation under control and exchange rates from devaluation. The risk that the Eurozone will end up in a deflationary zone is very possible, where accelerated inflation might be accompanied by a higher unemployment rate. This conflict delivered an immediate effect on the oil and gas prices and simultaneously exacerbates the food security problems. The likelihood that the conflict might expand to the other EU member states made the euro collapse against the US dollar. This devaluation is mainly driven by the investor's panic and the capital outflow from the Eurozone toward the US. In this context [1], analyzed the influence of the Russian ruble on the euro devaluation during the first three months of the war in Ukraine. Even though the ruble is a weak currency, the Eurozone's dependence on Russian gas has caused the devaluation of the euro. To this end, even weak currencies can outperform hard ones depending on the circumstances. Another study by Ref. [16] highlights that higher energy prices due to the conflict in Ukraine caused the strengthening of the Canadian dollar against the euro and the Japanese yen. After all, it was clear that the effects of this war would be mostly felt in the European territory [17]. consider that the economic consequences of this conflict will affect Germany the most, followed by France and Italy. To reinforce this argument [18], document that global economies nowadays are more vulnerable to international conflicts. From a financial perspective, issues such as social unrest, political instability, and various crises are easily transmitted to other countries. Sokhanvar et al. (2023) [19] investigated the surge in energy prices from the outbreak of the war in Ukraine through the 4-h time frame data between January and November 2022. Their findings indicate that the Australian dollar regained its position in FX during this period compared to the Euro, the Japanese yen, and the British pound. On the other hand, Hossain et al. (2023) [20] document that geopolitical risk due to the conflict in Ukraine has mainly exposed fragile democracies, weak economies, and those with heavy reliance on Russian gas. Moreover, they suggest that the most significant impact appeared in the returns experienced by capital markets as a result of capital flows.

The V4 countries moved from a centralized economy to a free market where the management of national currencies was the exclusive responsibility of the state central banks. The transition to a free-market economy made the national currencies of these countries subject to the market mechanism. The Czech crown (CZK), Polish zloty (PLN), and Hungarian forint (HUF) do not stand among hard currencies. They are not frequently traded in FOREX, and for this purpose do not hold the focus of speculators and institutional investors. Instead, currency sustainability is essential for keeping their economies viable in terms of foreign trade and inflationary pressures. Poland, the Czech Republic, Hungary, and six other countries in the bloc joined the EU on May 1, 2022. Since that time, they handed over part of the economic sovereignty to the EU but kept the monetary policy. Even though these countries are part of NATO, the conflict in Ukraine raised security alarms. The fact that the V4 countries are heavily reliant on Russian gas exacerbates the complexity of this problem. On May 5, 2022, Russia announced the "unfriend list of countries and territories", which includes the Czech Republic, Poland, and Hungary [21]. This means that the countries on the list must make gas payments in the ruble, which were previously made in euros. This made the Russian ruble quickly regain its lost position in FOREX and strengthen against the euro. Clearly, the sanctions imposed by Western countries would send the Russian ruble into free flow. However, the study by Ref. [22] confirms the turbulence the Russian Ruble has caused in FX markets. The dependence of the Czech Republic and Hungary on Russian gas exceeds 80 % while Poland stands at the level of 55 %. Presently, Poland plans to completely cut off the gas supply from Russia, as it will use liquefied natural gas (LNG) through the port of Gdańsk [23]. The reality regarding gas supply from Russia is not identical in the entire V4 countries. For this purpose, this study investigates if this request from Russia impacted the Czech crown, the Polish zloty, and the Hungarian forint. The findings of this article are valuable not only for policymakers but also for financial investors in general. Currency investors might consider the geopolitical context in addition to financial and economic factors. Recognizing the importance and seriousness of the situation, the authors present two objectives. The first one examines the effects of the Russian ruble on Czech crown, Polish zloty, and Hungarian forint. The second identifies the shocks that these pairs hold with each other. To address this issue, two research questions were constructed that correspond to each objective respectively.

Q1 *What is the importance of the Russian ruble in the changes that have taken place in the Czech crown, the Polish zloty, and the Hungarian forint?*

Q2 *What is the causal relationship between these four FX rates during the Russia-Ukraine war?*

The rest of the paper is structured as follows. Section 2 indicates the literature review and the context of the situation while the data and methods used are presented in Section 3. The results and discussions are placed in Section 4 while the concluding remarks in Section 5.

## 2 Literature and theoretical background

The exchange rate stability nowadays is carefully analyzed, considering the increased intensity of world trade and the growth of cross-border transactions. In addition to traditional economic factors, FX rates are subject to the fragility of democracies, the rule of law, and the degree of corruption. Surprisingly [24], document that exchange rate volatility is lower in high-corruption countries than in low-corrupt ones. The coronavirus disease and its rapid spread caused serious consequences for the foreign exchange rate market [25]. As a result of frequent interventions by national central banks, FX rates were standing on artificial bounds. According to the World Health Organization [26], the first case appeared on December 31, 2019, in the province of Wuhan, China. A few weeks later, the infection quickly spread all over China, and after three months global pandemic was declared. To prevent the infection, many countries took stringent measures that were unconstitutional and often in conflict with human rights. Due to restrictions on individual mobility and lockdown, the most affected sectors were aviation and tourism [27]. It was clear that government measures would have a counter-effect on economic growth [28], national consumption [29], and the financial system [[30], [31], [32]]. Due to restrictions, the global output crashed by 8.5 trillion $, pushing 34 million people into poverty [33]. In the first quarter of 2022, the World Trade Organization (WTO) declared that global trade shrank by 18.5 %. At the same time, equity and currency markets went into free fall, creating an unprecedented panic among investors [34]. This period was characterized as well by frequent instabilities in the FX rates mainly due to the disruptions in supply chains and expansive monetary policies [35]. using GARCH models analyzed exchange rate volatility during the COVID-19 pandemic. Their results claim that infection rate and death numbers have significantly affected the USD/EUR, USD/RMB, and USD/GBP. Emerging economies on the other hand, due to fiscal constraints, were limited to intervene in the economy. In this context [36], document that BRICS countries (Brazil, Russia, India, China and South Africa) put pressure on their currencies parity through low-interest rates. From a fiscal perspective [37], analyzed government measures in 20 countries and their consequences for the FX markets. They highlight that the infection rate and government actions carried further uncertainties in foreign exchange rates.

The globalization of financial markets together with deregulation has made the world financial system highly interdependent [[38], [39], [40]]. Foreign exchange markets are not excluded from this principle, where shocks in one currency inevitably give side effects to others. The standard finance textbooks emphasize that changes in the FX rates are subject to trade relations, financial crises, and capital flows. In this context, easily accessible information's is vital for investors' returns and their positions in the marketplace. The efficient market hypothesis confirms that investors take decisions considering all available information [41,42]. Although these principles are hardly achieved since asset prices follow a random walk. The euphoria, panic, and geopolitical circumstances in many cases overcome the investor's rationality. FX rates, like other financial securities, are not immune from these anomalies and phenomena. Moreover, exchange rates are exposed to continuous speculation and bubble formation. Instabilities in the FX market exacerbate market risk, shift international trade, and boost central banks' vigilance [43]. analyzed the impact of the Russian invasion of Ukraine for seven major international FX pairs. Their findings show that the Euro and the Swiss Franc have served as a hedge for currency portfolios. The crisis periods show that not only the FX market, but the entire financial system absorbs international effects [8,44,45]. Due to the interconnectedness between national economies and the financial system in general, exchange rates are prone to these movements [46,47]. The financial downturn of 2008/09 and the Greek debt crisis of 2010/11 showed that the global financial system is highly interconnected. Greece, a country with a GDP of less than 1 % of the European continent, managed to put the entire EU financial system into cardiac arrest. Similarly, timely information is valuable input for correcting currency pairs that are beyond estimated equilibrium. As revealed by Ref. [48], information concerning terrorist attacks is quickly priced into currency exchanges. On the other hand, commodity prices are closely observed by retail investors, financial institutions, and regulatory authorities. The work by Ref. [49] using neural networks documents that Brent crude oil has significantly influenced the EUR/USD parity. Another study during the 2008 crisis explored that the shocks to the US dollar directly transmitted the oil prices [50]. Recently, a study by Ref. [51] analyzed the impact on the Canadian dollar, the euro, and the Japanese yen that gas, oil, and wheat held during the conflict in Ukraine. Considering the period from February 1 to April 30, 2022, results document the spillover effect from commodity prices toward three hard currencies. Unlike other studies, we analyze the impact that the conflict in Ukraine has had on the currencies of the V4 countries. Countries like Poland, Hungary, and Czech Republic are very close to the conflict zone, which makes them vulnerable to a possible Russian attack. During this period, these countries were facing double-digit inflation and are simultaneously dependent on Russian gas and oil.

Motivated by the other authors on the spillover effect that currency pairs generate during the crisis period. This study draws attention to the context of the Russia-Ukraine war and its consequences for the V4 exchange rates. The national currencies of the Czech Republic, Poland, and Hungary are closely linked to the Eurozone. [52], through event studies, highlight that EU currencies during this conflict experienced profound devaluation while Asian ones strengthened their position in the FX market. Apart from gas and oil, these countries' trade relations with Russia are very limited. However, oil and natural gas are an integral part of the V4 economies. Using Fractionally Integrated GARCH (FIGARCH) [53], argue that oil prices during the Russia-Ukraine war caused a tremendous shock on the G7 currency pairs. The increase in gas prices or the possible supply cut-off by Russia might harm their competitive position. To complicate further the situation, since the beginning of June 2022, Russia requires gas payments to be made in rubles. Apart from the fact that the ruble remains the official currency of the Russian Federation, it is also used in the militarily occupied regions of Ukraine and Georgia. Despite the crash experienced at the beginning of the conflict in Ukraine, many daily newspapers considered the ruble as the currency with the best performance in 2022. This performance and recovery of lost value resulted from subsequent actions of the Russian government. The first and perhaps most important was the demand for gas payments in rubles. Second, the increase in the interest base rate to the level of 20 %. And finally, the actions related to the cross-border financial control of remittances. Compared to other studies, this is the first one that deals with the impact of the Russian ruble on the currency pairs of the V4 countries. Taking all these into account, the expectation is that the ruble might have produced currency devaluation in these countries. Despite the heating season, the energy crisis in Europe is not intensifying and the price of gas on the wholesale market is falling. This fact, among other things, raises alarm signals that energy dependence on autocratic regimes may have unforeseen economic consequences. Beyond the effects on international trade, changes in exchange rates also affect debt denominated in foreign currencies. Since joining the EU in 2004, the V4 countries have significantly raised their debt in foreign currency. Although most of the national debt is in local currency, a significant part is in Euro. The external debt of the Czech Republic in 2023 reached 185 million Euros, that of Poland close to 370 million, while that of Hungary at 150 million [54]. In this sense, small changes in the exchange rate can increase the fiscal burden and servicing the debt more difficult..

**Fig. 1:**
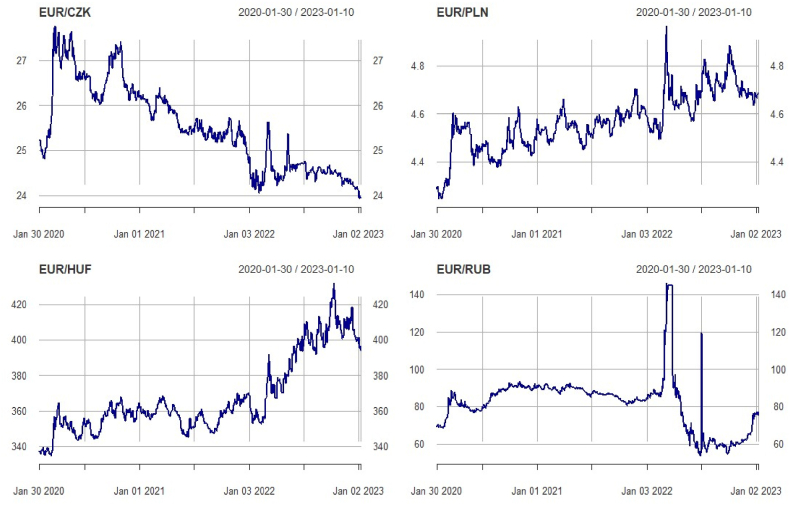
Presents the daily changes in the exchange rates of the V4 countries FX rates. **Note:**Fig. 1 contains four Euro FX rates based on the level data from January 30, 2020, to February 1, 2023. The EUR/CZK represents the pairs between the Euro and the Czech crown, EUR/RUB between the Euro and the Russian ruble, EUR/PLN between the Euro and the Polish zloty, and EUR/HUF stands for the Euro and the Hungarian forint. Plots are prepared in R studio using the *"ggplot2"* and *"tidyverse"* packages. Each FX rate displayed in the figure holds an identical number of observations (726). The x-axis represents the time frame, while the y-axis represents the convertible price of the currency against the Euro. Source: Authors own creation.

Fig. 1 presents the daily changes in V4 currency pairs from January 30, 2020, to February 1, 2023. The Czech crown in the first two months of COVID-19 lost 8 % of its value against the euro. The trend reversed after that time, where on February 1, 2023, CZK was traded at 1:23.7 with the euro. The Polish zloty went through devaluation as well and the problem further accelerated during the Russia-Ukraine war. The Hungarian forint from April 2020 to March 2022, oscillated between the 340 and 370 range. The Russian invasion of Ukraine caused a 14.8 % devaluation of the Hungarian forint toward the euro. Meanwhile, these countries are facing high inflation that has not been seen since the beginning of the 90s. In Hungary, core inflation in October 2022 has reached the level of 22.3 %, followed by the Czech Republic at 14.6 %, and Poland at 11 % [55]. In response to inflationary pressures, the Central Banks of these countries increased the interest base rate. The Hungarian Central Bank was more aggressive, setting the base rate at 13 %, followed by the Czech and Polish rates at 7 %. Despite the increase in interest rates, the Polish zloty and the Hungarian forint continued their devaluation. In the case of the Czech Republic, after the second intervention, the Czech crown started to recover. The reduction of state deficit during this period further strengthened the position of Czech crown. Future studies might put into play the interest base rate as an additional element in the analyses.

Considering the economic and security implications of this conflict for the European continent, this paper differs from the previous one in what follows. First, it treats the currencies of EU countries with national monetary policy while located at a short distance from the war zone. Second, these countries (Czech Republic, Poland, and Hungary) maintained heavy reliance on Russian gas and oil, which increases the significance of the findings. Although Russian gas and oil are not part of the direct analysis, they are analyzed through circumstantial. Third, the decree of the Russian president that gas payments must be made in rubles. All these facts and others (such as capital flights to the US during this period) provide reliable signals for state authorities, central banks, and currency investors. All these facts and others (such as capital flights to the US during this period) provide reliable signals for state authorities, central banks, and currency investors. All these facts and others (such as capital flights to the US during this period) provide signals for state authorities, central banks, and currency investors. These countries faced double-digit inflation during this period, where the currency downfall could accelerate this problem. The Czech, Polish, and Hungarian Central Banks were mitigating inflationary pressures as well as currency instability issues. This required shifting base interest rates to a higher level, with a clear counter-effect on economic growth. On the other hand, non-economic shocks (such as COVID-19 and the war in Ukraine) offer narrow diversification space for currency investors.

## 3 Methods

This section is divided into two parts, where Section 3.1 present the data collection and processing while 3.2 the methods used.

### 3.1 Data

This study investigates the impact of the Russian ruble on the Czech crown, Polish zloty, and Hungarian forint. The pairs used for analysis are EUR/CZK, EUR/PLN, EUR/HUF, and EUR/RUB, covering eight months of Russia-Ukraine war from February 1, 2022, to February 1, 2023. The series were collected by the "quantmod" package in R studio. Each pair contains 785 observations based on daily frequency. The Euro as a hard currency is attached to other pairs, which enables easier comparison.

**Fig. A.1:**
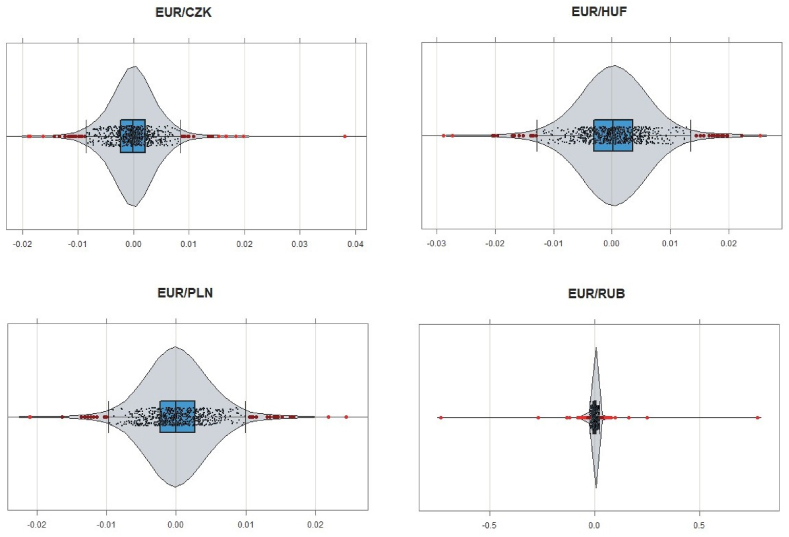
Indicates the violin boxplot distribution of the four-euro FX rates. **Note:** the figure displays the values of a violin boxplot distribution Deepayan Sarkar's lattice package in R studio. The scatterplots stand for the parities EUR/RUB, EUR/CZK, EUR/PLN, and EUR/HUF. The series covers the full period from February 1, 2022, to February 1, 2023. Each FX rate in the figure is performed based on daily logarithmic returns. The figure was prepared in R studio using the *"ggplot2"* package and the *"Plot"* function. The plots highlight minimum, maximum, lower and upper whisker, outliers, and median. The largest number of outliers stand for EUR/RUB due to the deep decline at the beginning of the conflict and then immediate recovering. Source: Authors own creation.

**Fig. A.2:**
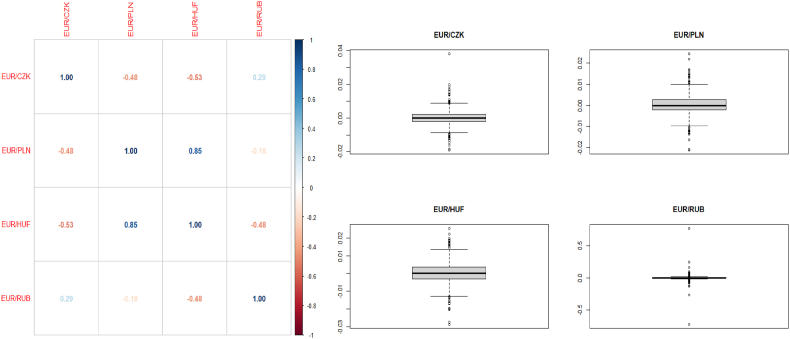
Display the correlation matrix and boxplot distribution of the four euro FX rates. **Note:** this figure stands for the correlation matrix of the four FX rates and their distribution through boxplots. The distribution of series in the boxplot stands on log returns, while the correlation matrix is ​​based on level data. Plots were performed in R studio using the *"ggplot 2″* package and the *"corrplot"* and *"boxplot"* functions. The series used to generate correlation matrix and box plots cover the full period from February 1, 2022, to February 1, 2023. Source: Authors own creation.

Table 1 presents the descriptive statistics of the four FX rates, such as EUR/CZK, EUR/PLN, EUR/HUF, and EUR/RUB. The results cover the full period from February 1, 2022, to February 1, 2023. Descriptive statistics are prepared on level data, while all other tests were conducted with daily logarithmic returns. Based on the Jarque-Bera test, the series do not possess normal distribution except for EUR/PLN. The maximum range stands for the EUR/HUF and EUR/RUB since they operate in higher parity and maintain the highest standard deviation (Sd). As for the kurtosis of our series, EUR/RUB is identified as leptokurtic (>3) while other FX rates as platykurtic (<3). The skewness highlights that EUR/PLN and EUR/CZK are symmetrical while EUR/RUB and EUR/HUF are highly skewed. The violin boxplot distribution of the series based on logarithmic returns is as well presented in Figure A1 in the appendix. Additionally, Figure A2 in the appendix shows the correlation matrix and the distributions through boxplots. The distribution of both histograms and boxplots is based on the daily logarithmic returns. The strongest positive correlation was identified between EUR/HUF and EUR/PLN (0.85), while the weakest was between EUR/RUB and EUR/PLN (−0.18). Before using the VAR model, the stationary of the data is of particular importance. The Augmented Dickey-Fuller Test (ADF), Phillip-Peron Test (PP), and Kwiatkowski–Phillips–Schmidt–Shin (KPSS) Test were used. Series at the level do not pass stationary tests but only after their logarithmic transformation.

**Table 1:** Indicates the descriptive statistics based on raw data.

	EUR/RUB	EUR/CZK	EUR/PLN	EUR/HUF
N	785	785	785	785
Min	53.71	23.77	4.24	334.64
Max	145.90	27.76	4.96	431.82
Mean	82.59	25.59	4.56	365.34
Median	85.99	25.45	4.56	359.08
Skewness	1.09	0.35	0.05	1.17
Kurtosis	5.15	−0.89	0.38	0.61
Sd	14.53	0.92	0.12	19.77
Range	92.19	3.71	0.72	97.17
JB test	0.000	0.000	0.087	0.000

**Notes:**Table 1 indicates descriptive statistics of the four FX rates based on the daily frequencies. The data covers the full period from February 1, 2022, to February 1, 2023. The items in the table stand for the number of observations (n), minimum (Min), maximum (Max), Median, Skewness, Kurtosis, standard deviation (Sd), Range, and Jarque Bera test (JB). The outcomes were generated in the R program using the *“tseries”* and *“lessR”* packages. Source: Authors own creation.

**Fig. 2:**
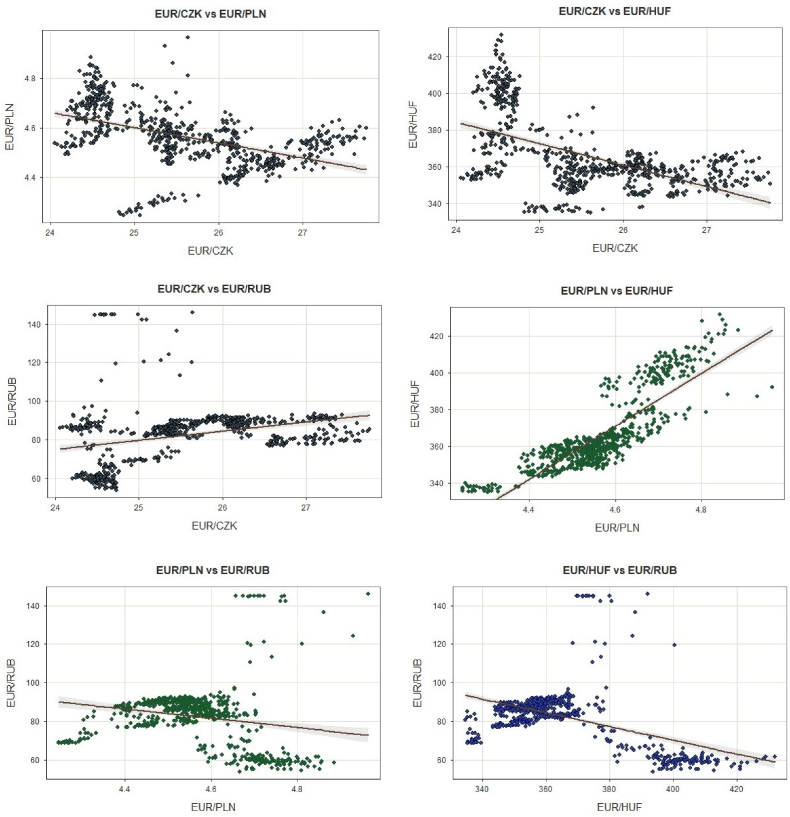
Pearson's product-moment correlation. **Note:** This figure presents Pearson's product-moment correlation based on raw data. Each plot indicates possible combinations (which, in our case, are six) between FX pairs in the system. Based on daily frequencies, the series covers the entire period from February 1, 2022, to February 1, 2023. The data points in each plot show the daily movement of exchange parities, while lm is used to fit a linear model. The x-axis contains the FX pair of one currency, while the y-axis contains the FX pair of another currency where the Euro is always the base unit. Source: Authors own creation.

Fig. 2 highlights combination pairs standing on Pearson's product-moment correlation with a 95 % confidence band. Two of the combinations maintain inverse relationships (those between EUR/CZK vs. EUR/PLN and EUR/CZK vs. EUR/HUF), one with a positive (EUR/PLN vs. EUR/HUF), and three with an unclear tradeoff (EUR/CZK vs. EUR/RUB, EUR/PLN vs. EUR/RUB, and EUR/HUF vs. EUR/RUB). Always considering that these relations represent only one year of the Russia-Ukraine war. In this context, the devaluation of the CZK toward the Euro has caused PLN and HUF to strengthen simultaneously. On the other hand, the appreciation/depreciation of EUR/HUF has had the same effect on the EUR/PLN parity. In contrast, the relationship is undefined for all the combinations where the Russian Ruble is present.

### 3.2 Methods

This work examines the effect that the Russian ruble has on the national currencies of the three Visegrad countries. The unrestricted VAR, Granger test, impulse response function, variance decomposition, and the Vector Error Correction Model (VECM) were used to generate the results. The Vector Auto Regression (VAR) is a multivariate time series model where the variables are influenced by their lags and the lags of other variables in the system. The VAR is a special type of the vector autoregressive moving average (VARMA) model where moving average lags are also used in its structure. The VAR model finds application in the analysis of monetary actions, fiscal policies, and economic crises. VAR is used as a forecasting technique as well by international financial institutions through its fan chart function. In unrestricted VAR, all variables are endogenous, and no exogenous variable exists in the system. The implementation in R studio was done through the packages *"vars", "tseries", "quantmod", "stargazer", "ggplot2", "plotrix",* and *"tsDyn"*. Before we implement the VAR model, the data must be tested for their unit roots. The ADF, PP, and KPSS in R studio were performed through the functions *adf. test (), pp. test (), and kpss. test ().* The exchange rate series generally do not pass the stationary test at the level but only after their logarithmic transformation. Determining the optimal number of lags is an additional element in constructing the unrestricted VAR. The lags (p) selection criteria in R are generated via the *"vars"* package and the *"optimal_lag$selection"* function. The unrestricted VAR consists of K endogenous variables $y_{t}=\left(y_{1t},y_{2t},\ldots ,y_{kt},\ldots ,K\right)$ where k=1,…,K. After including the number of lags, the unrestricted VAR (p) stands as follows.

(1) $$y_{t}=A_{1}y_{t-1}+\ldots +A_{p}y_{t-p}+{CD}_{t}+u_{t}$$

The $A_{i}$ in equation (1) indicates the coefficient matrices (KxK) which can take the form i=1,2,3,…,p, and $u_{t}$ based on the white noise process. The covariance dimensional (CD) matrix with a white noise process in it, takes the following form.

(2) $$\in \left[u_{t}\right]=0$$

(3) $$\in \left[u_{t}u_{t}^{\prime }\right]=\sum u$$

Equation *(2, 3)* clearly shows that the VAR (p) relies on variable (k), vector ($y_{t}$), and autoregressive lags. The following equation represents VAR (1) with four inputs and one lag autoregressive in the system.

(4) $$y_{t}=A_{1}+A_{11}y_{t-1}+A_{12}x_{t-1}+A_{13}z_{t-1}+A_{44}b_{t-1}+u_{t}$$

(5) $$x_{t}=A_{2}+A_{21}y_{t-1}+A_{22}x_{t-1}+A_{23}z_{t-1}+A_{44}b_{t-1}+g_{t}$$

(6) $$z_{t}=A_{3}+A_{31}y_{t-1}+A_{32}x_{t-1}+A_{33}z_{t-1}+A_{34}b_{t-1}+n_{t}$$

(7) $$b_{t}=A_{4}+A_{41}y_{t-1}+A_{42}x_{t-1}+A_{43}z_{t-1}+A_{44}b_{t-1}+e_{t}$$

The $u_{t}$, $g_{t}$, $n_{t}$, $e_{t}$ within equations (*4, 5, 6, 7*) stand for the shocks in the system while $y_{t}$, $x_{t}$, $z_{t}$, ${and\;b}_{t}$ represent the order of variables. In this case, we have four variables in the system that are constrained by one autoregressive lag. So, $y_{t-1}$, $x_{t-1}$, $z_{t-1}$, and $b_{t-1}$ in equation *(8)* indicate the autoregressive lags (p) of each variable within the VAR (1) model. This system of equations can also be expressed in the form of a matrix.

(8) $$\left[\begin{array}{c} y_{t}\\ x_{t}\\ z_{t}\\ b_{t} \end{array}\right]=\left[\begin{array}{c} a_{1}\\ a_{2}\\ a_{3}\\ a_{4} \end{array}\right]+\left[\begin{array}{c} a_{11}\;a_{12}\;a_{13}a_{14}\\ a_{21}a_{22}a_{23}a_{24}\\ a_{31}a_{32}a_{33}a_{34}\\ a_{41}a_{42}a_{43}a_{44} \end{array}\right]\;\left[\begin{array}{c} y_{t-1}\\ x_{t-1}\\ z_{t-1}\\ b_{t-1} \end{array}\right]\;\left[\begin{array}{c} u_{t}\\ v_{t}\\ l_{t}\\ e_{t} \end{array}\right]$$

The VAR is a model that originates from Ordinary Least Squares (OLS) where each equation performs independently. We have used four information criteria for determining the number of lags, such as Akaike Information Criterion (AIC), Hannan-Quin (HQ), Schwarz (SC), and Akaike Final Prediction Error (FPE). The VAR results are visualized through impulse response function (IRF) and variance decomposition (FEVD). In R studio, IRF and FEVD are generated with the "vars" package and implemented through the "irf" and "fevd" functions. On the other hand, the robustness test was conducted in the group through the Granger Casualty test.

In addition to unrestricted VAR, we have as well implemented the VECM. In the R program, VEC Model is generated by the "vars" package and the "vecm" function. The implementation of the VEC Model requires that variables in the system must hold at least one co-integration. The co-integration relation is formed when two or more variables in the system maintain long-run association [56]. The Johansen Co-integration test in R is implemented through the "urca" package and the "ca.jo" functions. The mathematical formula for the Johansen test takes the following form.

(9) $$s_{v}=A_{1}s_{v-1}+e_{v}$$

Where

(10) $$\Delta s_{v}=A_{1}s_{v-1}-\;s_{v-1}+e_{v\;}$$

(11) $$=\left(A_{1}-I\right)s_{v-1}+e_{v}$$

The vectors on each equation are denominated with $s_{v}$ and $e_{v}$ while the trace and eigenvalue matrix are presented with $A_{1}$. In case we have four variables, then a maximum of four independent vectors might be in the system. Based on the variables we have in the system, the sequential tests can take the form *0, 1, 2, 3, 4, 5, 6 … n*. The rank of 0 represents no co-integration in the system, 1 vector indicates 1 co-integration, and so on. The Johansen Co-integration test in R studio is executed using trace statistics and maximal eigenvalue. Once Co-integration relations were identified in the system, then it is possible to continue with the VECM implementation. As with VAR, determining the optimal number of lags within the VECM is important. The VECM is a special type of VAR for the time series that are stationary at their first difference. At the same time, VECM identifies the short-run and long-run causal relationship of the variables in the system.

## 4 Results and discussions

This Section consists of three parts, where Section 4.1 highlights VAR findings, Section 4.2 presents the outcomes of the VEC Model, and the robustness tests are placed in Section 4.3.

### 4.1 VAR estimation results

**Fig. A.3:**
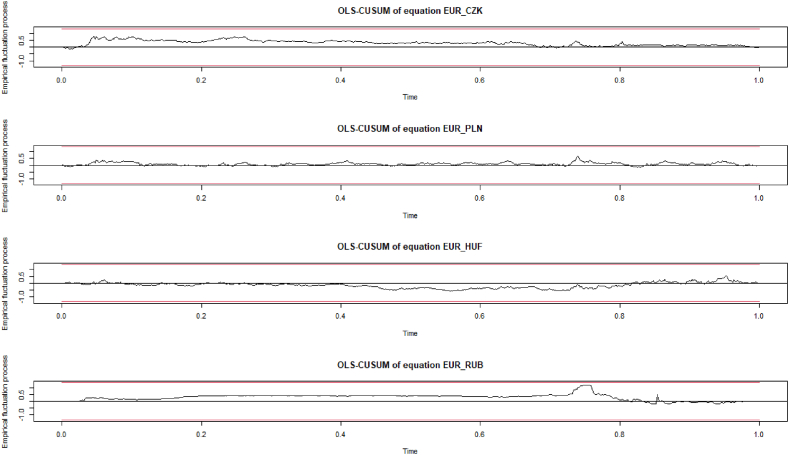
Structural breaks of the four-euro FX rate pairs used in our work. **Notes:** This figure presents structural breaks of the four-euro FX rates, such as EUR/CZK, EUR/PLN, EUR/HUF, and EUR/RUB. The figure was prepared in R studio through the *"vars"* package and the *"stability"* functions. The red lines represent the 95 % confidence band, while the black lines represent the movements of the residuals. The series stands on its logarithmic return and cover the entire period, from February 1, 2022, to February 1, 2023. Source: Authors own creation.

This study analyzes the effects of the Russian ruble on the Czech crown, the Polish zloty, and the Hungarian forint. In this context, results from unrestricted VAR (3), IRF, and FEVD were performed to analyze if this causality stands. The series do not pass the unit root test (ADF, PP, and KPSS) at the level, but only after transforming into the logarithmic return. Figure A3 in the appendix presents structural breaks which indicate a stable model since the residuals of the four variables lie within the 95 % confidence band. Three additional diagnostic tests were performed such as normality test, arch effect, and heteroscedasticity. The series does not pass the normality test, but they pass the serial correlation test and heteroscedasticity. The determination of the optimal number of lags was made through four information criteria (AIC = 3, HQ = 3, SC = 2, and FPE = 3). Since the most repeated number was three, then we have set three lags (l3) in the system.

**Fig. 3:**
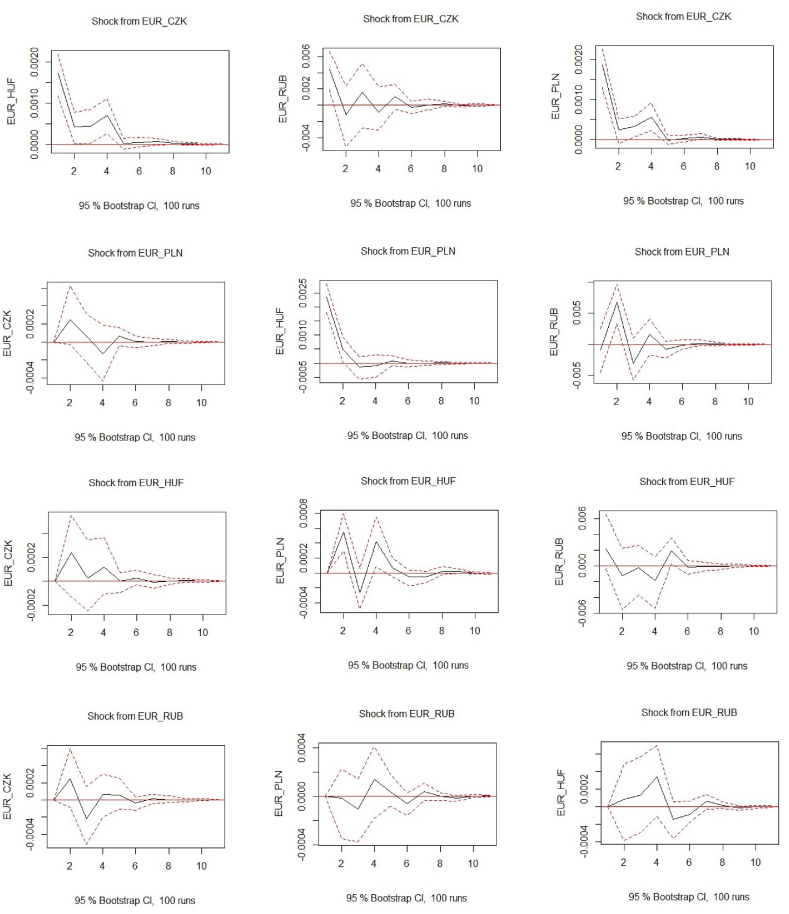
Indicates the VAR (3) impulse response function of the four-euro FX rates. **Note:**Fig. 3 highlights the VAR (3) impulse response function (IRF) with nine combinations between EUR/CZK, EUR/PLN, EUR/HUF, and EUR/RUB. The IRF shocks stand within 95 % bootstrap, simulated with 100 trials, and based on three lags. The shocks among each pair are constrained for the next 10 periods. Since our data stand on daily logarithmic returns, then the next 10 periods indicate the 10 trading days. The red lines within the plots present the error term while the black line the estimated shocks. The series covers the entire period from February 1, 2022, to February 1, 2023. The figure was created in R studio using the *"vars"* package, and the *"irf"* function, and visualized with the *"plot"* function. The horizontal line (x-axis) in each plot represents the time frame, which is ten days ahead. The shocks appearing with the percentage changes are on the vertical axis. Source: Authors own creation. (For interpretation of the references to colour in this figure legend, the reader is referred to the Web version of this article.)

Table 2 contains the results of the VAR (3) model with three lags in the system and based on the daily logarithmic return. Model (1) represents the impact that EUR/CZK has from its lags and lags of other FX rates in the system. We can conclude that EUR/CZK during this period is affected only by its lags (l1, l2, and l3). In Model (2) we see that EUR/PLN is influenced by lags of EUR/CZK (l2 and l3), EUR/HUF (l1, l2, and l3), and by its lags (l2 and l3). The EUR/HUF placed in Model (3) has received most of the influences from the EUR/CZK (l1 and l2) and its lags. In the end, EUR/RUB was mostly influenced by EUR/PLN (l1 and l3), EUR/CZK (l2), and its lags (l1 and l2). EUR/CZK has performed unaffected by the other FX rates but has influenced the movements of the others. On the contrary, EUR/RUB has not affected any of the FX rates but has received influence from EUR/CZK and EUR/PLN. From this, we can see that the Russian Ruble has received influence from CZK and PLN but has not influenced them.

**Table 2:** The VAR (3) of the four-euro FX rates log return series.

Variables	Model (1)	Model (2)	Model (3)	Model (4)
	EUR/CZK	EUR/PLN	EUR/HUF	EUR/RUB
EUR/CZK.l1	−0.155*** (0.041)	0.041 (0.042)	0.028 (0.054)	−0.485 (0.387)
EUR/PLN.l1	0.039 (0.044)	−0.062 (0.045)	0.082 (0.057)	1.611*** (0.409)
EUR/HUF.l1	0.043 (0.033)	0.106*** (0.034)	0.084* (0.043)	−0.062 (0.308)
EUR/RUB.l1	0.006 (0.004)	−0.0004 (0.004)	0.002 (0.005)	−0.440*** (0.037)
EUR/CZK.l2	0.073** (0.042)	0.141*** (0.043)	0.382*** (0.063)	0.207*** (0.047)
EUR/PLN.l2	0.006 (0.044)	−0.098** (0.045)	0.064 (0.057)	0.103 (0.412)
EUR/HUF.l2	0.008 (0.033)	−0.057* (0.038)	−0.184*** (0.042)	−0.227 (0.306)
EUR/RUB.l2	−0.002 (0.004)	−0.003 (0.004)	0.004 (0.006)	0.211*** (0.005)
EUR/CZK.l3	0.115*** (0.041)	0.155*** (0.042)	0.0167*** (0.054)	−0.313 (0.387)
EUR/PLN.l3	−0.039 (0.044)	−0.126*** (0.045)	−0.013 (0.057)	0.852** (0.412)
EUR/HUF.l3	0.027 (0.033)	0.110*** (0.034)	0.012 (0.043)	−0.369 (0.309)
EUR/RUB.l3	−0.001 (0.004)	0.001 (0.004)	0.010* (0.005)	0.023 (0.037)
Const	−0.0001 (0.0002)	0.0001 (0.0002)	0.0003 (0.0002)	−0.001 (0.002)
Observations	782	782	782	782
*R*^2^	0.045	0.065	0.066	0.195
Adjusted *R*^2^	0.029	0.050	0.050	0.182
Residual Std. Error (df = 346)	0.004	0.004	0.006	0.041
F Statistic (df = 10; 346)	2.826***	4.220***	4.253***	14.624***

**Notes:** This table presents the estimated coefficients and standard errors of the unrestricted VAR (3). Each euro FX rates hold 782 observations out of 785 due to three lags in the system. The series stands in their logarithmic return and cover the full period from February 1, 2022, to February 1, 2023. ***, **, and * denote significance at the 1, 5, and 10 percent levels, respectively. Source: Authors own creation.

Fig. 3 presents IRF plots between EUR/CZK, EUR/PLN, EUR/HUF, and EUR/RUB based on daily logarithmic returns. The results stand in line with those presented in Table 2 where EUR/CZK remain unaffected but hold an influence on all other FX rates. In contrast to Tables 2 and in IRF plots the EUR/RUB is influenced by EUR/PLN and EUR/CZK. On the other hand, IRF again confirms that EUR/RUB does not affect other FX rates. Meanwhile, EUR/HUF once again influences only EUR/PLN and not the others..

**Fig. 4:**
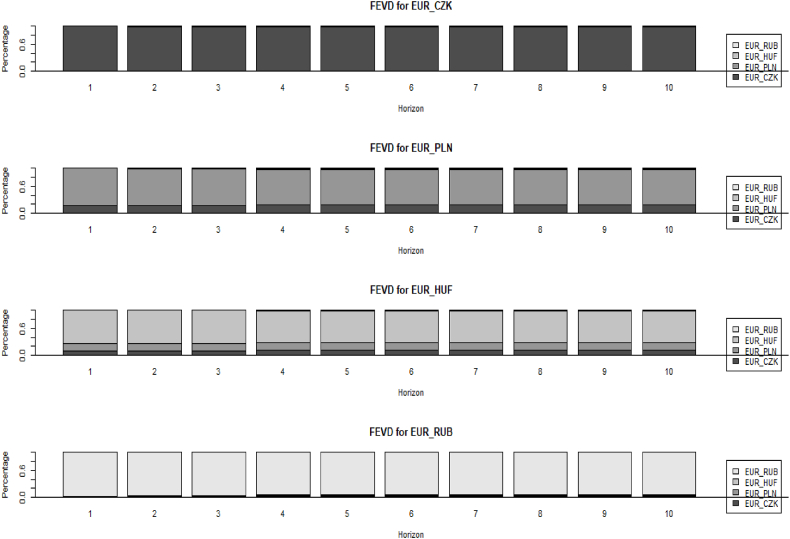
Presents the VAR (3) variance decomposition (FEVD) of the four-euro FX rates. **Notes:** This figure contains VAR (3) variance decomposition (FEVD) based on four-euro FX rates. The series covers the full period from February 1, 2022, to February 1, 2023, based on daily logarithmic returns. Meanwhile, the forecast variance error of each FX rate is constructed for the ten periods ahead. The figure was generated in R studio using the *"vars"* package and the *"fevd"* and *"plot"* functions. The horizontal line (x-axis) shows the period, which in our case is ten days ahead (since the data is daily). The vertical line (y-axis) represents percentage changes reaching 1 (or 100 %). Source: Authors own creation.

Fig. 4 presents FEVD for 10 consecutive trading days of EUR/CZK, EUR/PLN, EUR/HUF, and EUR/RUB. The EUR/CZK in the next ten trading days is not affected by any of the other FX rates. On the other hand, EUR/PLN, from the first day to the tenth day, is influenced by EUR/CZK between 17 and 18 %. The EUR/HUF from the first day to the tenth is influenced by EUR/PLN at the level of 17 %, while EUR/CZK is between 9 % and 11 %. In the end, the EUR/RUB moves almost alone from the influence of others for the entire period. Similarly, the significant impact of the Czech crown on the Polish zloty and the Hungarian is confirmed in FEVD as well. In the meantime, the Russian ruble remains without any influence on the other three pairs. Despite the fact that the Czech Republic is a smaller economy than Poland, the Czech crown appears to have a much greater influence. This is mainly due to the Czech Central Bank (CNB) actions, which was committed in preventing excessive currency devaluation during this period.

### 4.2 VECM estimation results

The VEC Model measures the short-term and long-term association between variables in the system. The model is implemented in R through the *"vars"* package and the *"vecm"* function. However, before VECM is implemented, the series must maintain at least one co-integration. For this purpose, the Johansen Co-integration test with trace statistics and maximal eigenvalue was implemented. The series in the system was performed using the maximum likelihood vector (ML). As for the number of lags, the information criteria have suggested the use of two lags in the system.

Table 3 presents the results of trace statistics and maximal eigenvalue standing on two optimal lags in the system. Both types of tests show that the test statistic (Test) is greater than the critical value with 1 %, 5 %, and 10 % significance levels. From this, we can conclude that in both tests (maximum eigenvalue and trace statistics) at least we have three Co-integration relations in the system. This confirms that we can continue with the implementation of the VEC model.

**Table 3:** Johansen Co-integration test with trace statistics and maximal eigenvalue.

Test type: trace statistic, without linear trend and constant in cointegration				
Eigenvalues (lambda):	[1] 0.418	[2] 0.403	[3] 0.353	[4] 0.276
Values of the test statistic and critical values of the test:				
	**Test**	**10 %**	**5 %**	**1 %**
r≤3	239.05	7.52	9.24	12.97
r≤2	561.41	17.85	19.96	24.60
r≤1	943.44	32.00	34.91	41.07
r = 0	1344.01	49.65	53.12	60.16
**Test type:** maximal eigenvalue statistic (lambda max), without linear trend and constant in cointegration				
Values of the test statistic and critical values of the test:				
	**Test**	**10 %**	**5 %**	**1 %**
r≤3	224.23	6.41	9.24	12.97
r≤2	322.02	13.75	15.67	20.20
r≤1	382.02	19.77	22.00	26.81
r = 0	400.58	25.56	28.14	33.24

**Notes:** This table indicates the Johansen Co-integration tests with trace statistics and maximal eigenvalue covering the four euro FX pairs. The results stand on daily logarithmic returns covering the full period from February 1, 2022, to February 1, 2023. The results are performed in R studio through the *"urca"* and *"vars"* packages. Source: Authors own creation.

Table 4 presents the results of the VEC Model with frequencies from February 1, 2022, to February 1, 2023. The EUR/CZK is significant at 1 % in ECT1 and ECT3, where the long-term disequilibrium is corrected at the level of 43.1 % and 26.3 %, respectively. Even in EUR/PLN, most of the long-term corrections occur on the first day (71.2 %). The same applies to EUR/HUF which maintains a 1 % significance level in ECT1 and ECT3 where most of the corrections occur. In the end EUR/RUB, where 76.1 % of corrections occur in ECT2, i.e., on the second day. Since our data is daily, then most corrections of long-term disequilibrium occur in the first trading days. In the short term, the VECM results indicate different behavior of series in the system. The EUR/CZK in the first and second lag (l1 and l2) significantly influences (at 1 %) itself, EUR/PLN, and EUR/HUF. The EUR/PLN appears to have an impact only on itself in the first and second lag (l1 and l2) at a 10 % significance level. The EUR/HUF (l1 and l2) affects EUR/CZK and EUR/PLN at a 5 % significance level. Finally, EUR/RUB does not affect any other pairs except itself (l1). After all, even in the VEC, the results show a strong influence of the Czech crown on other pairs while no influence of the Russian ruble. In contrast to VAR (3), vector error correction results show that the Russian ruble does not absorb any impact from other currencies.

**Table 4:** Presents the estimated results of the error correction model of the four FX rate series.

	EUR/CZK	EUR/PLN	EUR/HUF	EUR/RUB
r1	1.000	0.000	(0.000)	(0.715)
r2	(0.000)	1.000	(0.000)	(0.626)
r3	(0.000)	0.000	1.000	(0.828)
**Equation**	**ECT1**	**ECT2**	**ECT3**	**Intercept**
EUR/CZK	−0.4308(0.0715)***	0.1370(0.0896)	0.2637(0.0616)***	−0.0001(0.0002)
EUR/PLN	0.7119(0.0696)***	0.1937(0.0872)***	0.2898(0.0599)***	0.0001(0.0002)
EUR/HUF	0.8207(0.0888)***	0.2516(0.1113)*	−0.9187(0.0765)***	0.0002(0.0002)
EUR/RUB	0.3598(0.6134)	0.7614(0.7686)***	−0.3798(0.5281)	−0.0005(0.0015)
**Equation**	**EUR/CZK.l1**	**EUR/PLN.l1**	**EUR/HUF.l1**	**EUR/RUB.l1**
EUR/CZK	−0.5565(0.0605)***	−0.0463(0.0756)	−0.1550(0.0486)**	0.0025(0.0076)
EUR/PLN	−0.5542(0.0589)***	0.1685(0.0687)*	−0.1381(0.0473)**	0.0018(0.0074)
EUR/HUF	−0.6404(0.0752)***	−0.1230(0.0877)	0.00615(0.0603)	−0.0135(0.0094)
EUR/RUB	−0.5964(0.5190)	−1.0730(0.6052)	0.41474(0.4167)	0.2335(0.0650)***
**Equation**	**EUR/CZK.l2**	**EUR/PLN.l2**	**EUR/HUF.l2**	**EUR/RUB.l2**
EUR/CZK	−0.2751(0.0427)***	−0.0064(0.0480)	−0.1046(0.0353)**	0.0002(0.0043)
EUR/PLN	−0.2670(0.0415)***	0.0942(0.0467)*	−0.1642(0.0344)***	−0.0015(0.0042)
EUR/HUF	−0.3129(0.0530)***	−0.0284(0.0569)	−0.0826(0.0439)	−0.0101(0.0054)
EUR/RUB	0.0740(0.3661)	−0.9191(0.4117)	0.2538(0.3032)	0.0222(0.0371)

**Notes:** This table presents the results of the error correction model related to four-euro FX rates. The series covers the full period from February 1, 2022, to February 1, 2023. The system is built on two autoregressive lags, and three co-integration vectors and is based on daily logarithmic returns. Since we possess three Co-integration relations, so we have three error correction terms in the system (ECT1, ECT2, and ECT3). The error correction term (ECT) shows how quickly the time series adjust their long-term imbalance. ***, **, and * denote significance at the 1, 5, and 10 percent levels, respectively. Source: Authors own creation.

The Granger test stands as a robustness test to identify whether one variable in the system predicts the changes of the other variables. Each FX rate is tested toward all other pairs based on their daily logarithmic returns. Table A1 in the appendix presents the Granger casualty test based on group testing. This stands as the limitation since the FX rates were tested in a group, while the results may be different if they would be tested individually. The p-value is less than 5 % in the case of EUR/CZK, EUR/PLN, and EUR/HUF, while higher than 5 % in the case of EUR/RUB. In other words, the Czech krona, Polish zloty, and Hungarian forint Granger cause each other in a group, but not the Russian ruble. So even in this test, the Russian ruble remains without influence on three other V4 currencies. The economic logic of these results also corresponds with the current reality. These countries (the Czech Republic, Poland, and Hungary) maintain intensive commercial, economic, and financial relations. Moreover, they are part of a common cultural, economic, and energy unit known as Visegrad and at the same time part of the EU. On the other hand, Russia, apart from gas and oil, is not considered the main component in the trade balance of these countries. The seaborn Russian oil is sanctioned by the EU, while the situation with gas is different. Poland has cut off gas supplies from Russia due to the use of liquid LNG in the port of Gdansk. While the Czech Republic and Hungary are supplied with gas from Germany, where the contracts are in euros, not in Russian rubles. Therefore, these results correspond with the existing situation and events during this period.

## 5 Conclusion

The Russian invasion of Ukraine created unprecedented panic in the European financial system and disrupted currency exchanges. The national currencies of the V4 countries went into cardiac arrest due to the geographical proximity to Ukraine and heavy reliance on Russian gas. The large flow of refugees and the possibility of Russian expansion into the EU territory endangered national security and accelerated inflation. On March 31, 2022, Russia announced that nations labeled "unfriendly" to the Russian government will make gas payments in rubles. The Czech Republic, Hungary, and Poland were added to this list in May 2022. On the other hand, Russian crude oil was banned by the EU, and the decision became effective on December 5, 2022. Standing on these facts, we investigate the impact of the Russian ruble on Polish zloty, Czech crown, and Hungarian forint. To this end, the purpose of this paper is twofold. First, is analyzed the impact of the Russian ruble throughout this period. Second, the influence that these currency pairs hold among themselves. All tests show that the Russian ruble has not maintained any influence on the Czech crown, the Polish zloty, and the Hungarian forint. The reasons are related to the fact that these countries make gas payments in euros. Since these countries are supplied with gas by Germany where contracts are denominated in euros. On the other hand, Poland terminated the gas supply from Russia after creating LNG gas reserves in the port of Gdansk. We can summarize that the EU managed to preserve a effects of gas supply but at the cost of high inflation and permanent nuclear risk.

An interesting result of this study is that the Czech crown is of particular importance in this region. The Czech crown during this period had significant influence on the Polish zloty and Hungarian forint. Tests from IRF, FEVD, and VECM show that the Czech crown is influenced by its lags while does not absorb effects from the Russian ruble, Polish zloty, and Hungarian forint. Other scholars can identify the reasons for the Czech crown's importance, which may not necessarily be economic. The reasons might be linked to the trade balance, national incomes, or confidence that the Czech economy reflects. Also, the fact that the Czech krona has strengthened its position might have boost investors' confidence and served as a benchmark for the Polish zloty and the Hungarian forint. However, the series covers only the period of the Russia-Ukraine war which may not be a complete picture of reality. Future studies may investigate longer periods and involve other currencies as well. The findings of this paper may be of interest to the central banks of these countries, national governments, and potential investors. From a policy perspective, the results inform the V4 governments that the Russian ruble was not a factor in the destabilization of V4 exchange rates. The motives might be related to capital flights from V4 countries towards the Eurozone or the USA.

## Additional information

No additional information is available for this paper.

## CRediT authorship contribution statement

**Florin Aliu:** Writing – original draft, Supervision, Software, Formal analysis, Conceptualization. **Jiří Kučera:** Writing – review & editing, Validation, Resources, Data curation. **Jakub Horák:** Writing – review & editing, Supervision, Methodology.

## Declaration of competing interest

The authors declare that they have no known competing financial interests or personal relationships that could have appeared to influence the work reported in this paper.

## Figures and Tables

**Table A.1:** Granger causality tests for the system of variables

Hypothesis testing	p-value	H0
Granger causality H0: EUR/CZK do not Granger-cause EUR/PLN, EUR/HUF, and EUR/RUB	0.0008	Rejected
Granger causality H0: EUR/PLN do not Granger-cause EUR/CZK, EUR/HUF, and EUR/RUB	0.0006	Rejected
Granger causality H0: EUR/CNY do not Granger-cause EUR/USD, EUR/JPY, EUR/GBP, and EUR/RUB	0.0004	Rejected
Granger causality H0: EUR/RUB do not Granger-cause EUR/CZK, EUR/HUF and EUR/PLN	0.4095	Accepted

**Notes:** This table shows the Granger causality test where each variable is tested against all other variables in the system. The five variables in the table cover the full period from February 1, 2022, to February 1, 2023. The tests were conducted on the logarithmic return series. The results are prepared in the R studio and analyzed through the *“urca”* package. Source: Authors own creation.
